# Mechanical characterization of nanoindented graphene via molecular dynamics simulations

**DOI:** 10.1186/1556-276X-6-481

**Published:** 2011-08-03

**Authors:** Te-Hua Fang, Tong Hong Wang, Jhih-Chin Yang, Yu-Jen Hsiao

**Affiliations:** 1 Department of Mechanical Engineering, National Kaohsiung University of Applied Sciences, 415 Chien Kung Rd., Kaohsiung 807, Taiwan; 2 Central Product Solutions, Advanced Semiconductor Engineering, Inc., Kaohsiung 811, Taiwan; 3 Institute of Mechanical and Electromechanical Engineering, National Formosa University, Yunlin 632, Taiwan; 4 National Nano Device Laboratories, Tainan 741, Taiwan

**Keywords:** molecular dynamics, nanoindentation, graphene, mechanical properties

## Abstract

The mechanical behavior of graphene under various indentation depths, velocities, and temperatures is studied using molecular dynamics analysis. The results show that the load, elastic and plastic energies, and relaxation force increased with increasing indentation depth and velocity. Nanoindentation induced pile ups and corrugations of the graphene. Resistance to deformation decreased at higher temperature. Strong adhesion caused topological defects and vacancies during the unloading process.

## Introduction

Graphene has received a lot of attention due to its good mechanical and electromagnetic properties [1-3], including a zero electron bandgap, a high electron emission rate, and elastic scattering [4-6]. Atomic-scale graphene can be fabricated using micro-mechanical chop crack [7], thermal expansion [8], and extension growth [9] techniques. Studies [10-13] have found that the band-field effect of a 10-nm-thick graphene sheet is similar to that of a small (less than 1.2 nm in diameter) nanographite particle.

Novoselov and Geim [7] used graphene to fabricate a small crystal tube. Monolayer graphene is considered a suitable material for investigating two-dimensional quantization phenomena, such as temperature-trigger plasma [14], quantization absorption spectrum [15], and the fractional quantum Hall effect [16]. In addition, the hexagonal symmetric structure of graphene makes it a candidate material for nano devices.

Many studies [17-25] have focused on the chemical functionalization of graphene, especially on the effect of absorbed atoms on the electronic and chemical properties of graphene. However, the mechanical properties of graphene under indentation, which are important for developing sensors, resonators, and impermeable membranes, have yet to be investigated.

In this study, the effects of nanoindentation depth and velocity on the mechanical properties and contact behavior of graphene at various temperatures are investigated using molecular dynamics (MD) simulations. Adhesion, relaxation, defects, and deformation are discussed.

## Methodology

**Figure 1:**
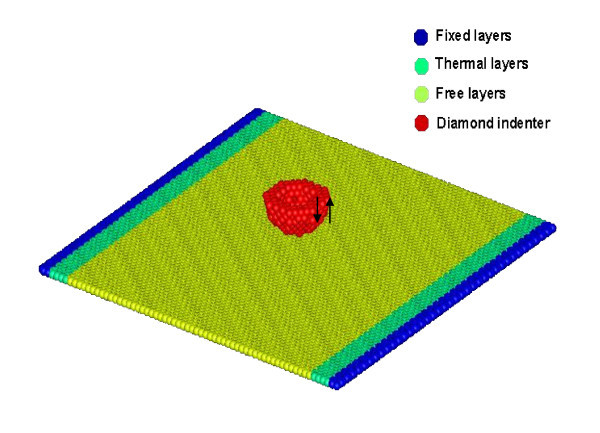
**Physical model of graphene substrate and indenter tip**.

Figure 1 shows the MD model of a freestanding honeycomb graphene sheet and a diamond indenter tip. The graphene substrate consists of 10,032 carbon atoms over an area of 15.874 × 15.933 nm. In the model, three layers of carbon atoms are fixed using a bridge-type support and six carbon lateral layers of thermostat atoms are set as thermal layers. The other carbon atoms are Newtonian atoms. The hemispherical diamond tip has 344 carbon atoms and is treated as a rigid body. The diamond indenter is 1 nm above the graphene surface; it approaches the graphene surface at a constant velocity.

The Lennard-Jones potential function is employed to describe the interaction between the diamond tip and the graphene atoms. The Tersoff empirical potential energy function [10] is generally used to simulate the interaction between graphene carbon atoms.

## Results and discussion

**Figure 2:**
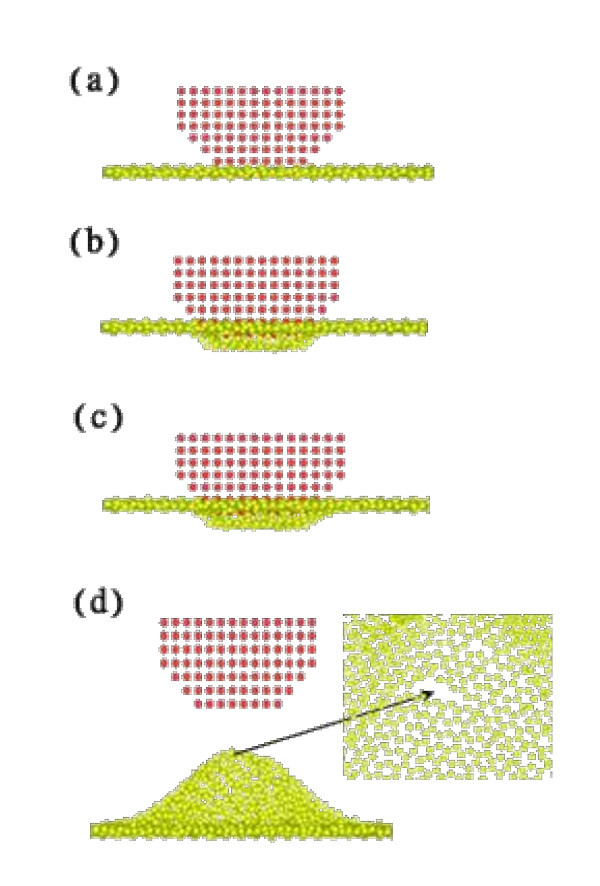
**Snapshots of indentation process**. At (**a**) 33 ps, (**b**) 44 ps, (**c**) 59 ps, and (**d**) 130 ps.

Figure 2 shows snapshots of graphene being indented by the hemispherical diamond tip at a velocity of 25 m/s, a hold time of 15 ps, and a temperature of 300 K. Thermal equilibrium was achieved before the indentation to have the atoms in a stable state. Figure 2a shows the initial contact of indentation at 33 ps. During indentation, the potential energy of the tip affects the surface atoms, especially those beneath the tip. Thus, some of the atoms jumped up and made contact with the tip, which is known as the jump-to-contact phenomenon.

The tip then indented the graphene. The absorptive force gradually turns into a repulsive force. As the indentation depth increased, the stress wave spread out farther from the center, inducing ripples and corrugations. Figure 2b shows the tip at its maximum indentation depth. During the subsequent packing stage, the substrate releases the indentation-induced energy, as shown in Figure 2c. Finally, the tip moves up at a constant velocity (the same as that used for the indentation). Some substrate atoms beneath the tip move up during the unloading process to create a peak, as shown in Figure 2d.

**Figure 3:**
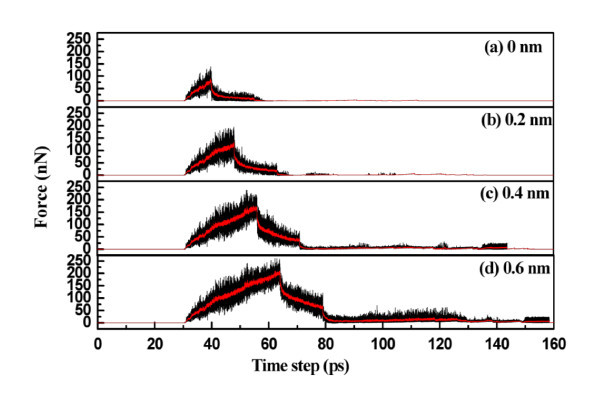
**Load versus time for various indentation depths**.

**Figure 4:**
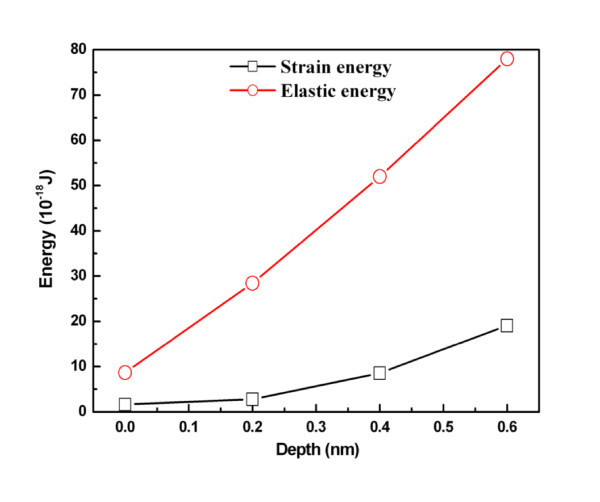
**Elastic energy and plastic energy versus indentation depth**.

The effect of indentation depth was examined. With all other conditions fixed, indentation depths of 0, 0.2, 0.4, and 0.6 nm were investigated. Figure 3 shows the force versus time curves for various indentation depths. The average maximum forces at indentation depths of 0, 0.2, 0.4, and 0.6 nm are 139.43, 195.47, 240.42, and 260.08 nN, respectively, indicating that the load increased with indentation depth. This is due to the number of atoms in contact with the tip increasing with indentation depth. Figure 4 shows the elastic energy and plastic energy versus displacement curves. The elastic and plastic energies both increase with increasing displacement. The average relaxation forces are 123.71, 141.10, 156.00, and 161.21 nN for indentation depths of 0, 0.2, 0.4, and 0.6 nm, respectively. The central heights of the residual ripple after unloading are 0.998, 1.104, 1.253, and 1.3224 nm for indentation depths of 0, 0.2, 0.4, and 0.6 nm, respectively.

**Figure 5:**
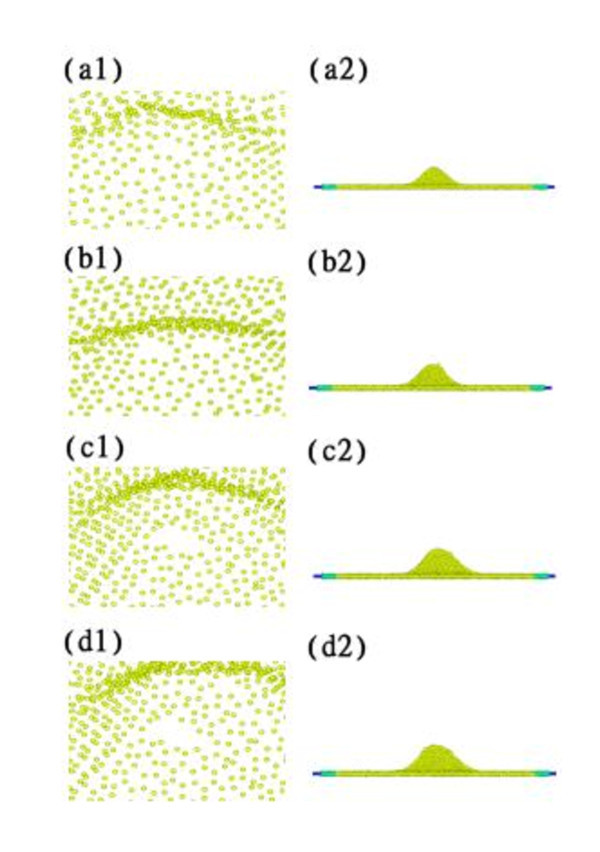
**Topographies for indentations**. Indentations of (a1, a2) 0 nm, (b1, b2) 0.2 nm, (c1, c2) 0.4 nm, and (d1, d2) 0.6 nm.

Figure 5 shows the topographies obtained for various indentation depths. The peak is larger for a deeper indentation due to more atoms adhering to the tip. Pile ups and corrugations of the graphene occurred beneath the indenter tip. The strong adhesion led to topological defects and vacancies. Stone-Wales defects usually play an important role in the corrugation region with 5-7-7-5 ring defects [26]. We also found double vacancy (C_2_) defects, which are composed of one octagonal ring and two pentagonal rings, during the adhesion pulling process. Double vacancies are referred to as 5-8-5 defects [27]. Our simulation results agree with those reported by Kudin et al. [28], who investigated the Raman spectra of graphite oxide and functionalized graphene sheets with Stone-Wales defects and C_2 _defects [28].

**Figure 6:**
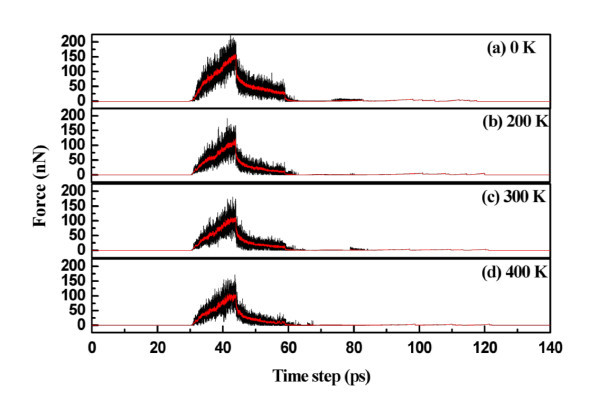
**Load versus time for various temperatures**.

Figure 6 shows the load versus time curves for temperatures of 0, 200, 300, and 400 K, respectively, with a velocity of 25 m/s and a packing time of 15 ps. At lower temperature, a higher force was required to achieve a given indentation depth due to the higher hardness of the material. The average maximum forces at sample temperatures of 0, 200, 300, and 400 K are 221.57, 191.24, 181.59, and 172.10 nN, respectively. Temperature control is thus necessary for a stable mechanical response. The average contact stiffnesses of the graphene at temperatures of 0, 200, 300, and 400 K are 58.7, 58.1, 49.48, and 36.6 N/m, respectively.

**Figure 7:**
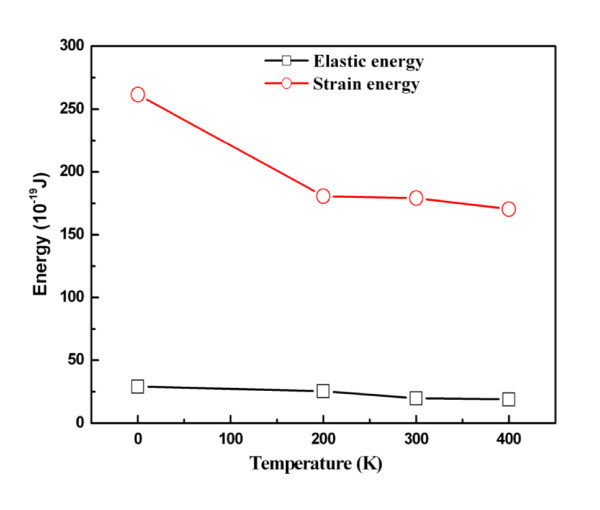
**Elastic energy and plastic energy versus temperature**.

**Figure 8:**
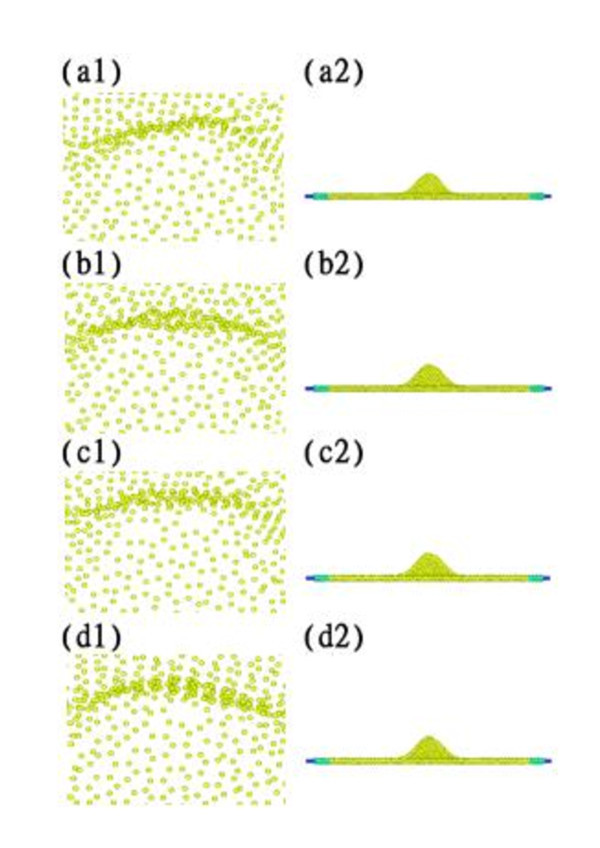
**Topographies for temperatures**. Tempeartures at (a1, a2) 0 K, (b1, b2) 200 K, (c1, c2) 300 K, and (d1, d2) 400 K.

Figure 7 shows the elastic energy and plastic energy versus temperature curves. Both energies decrease with increasing temperature due to the increasing distance between atoms. Reduced relaxation forces were calculated to be 176.25, 167.38, 159.705, and 152.14 nN for 0, 200, 300, and 400 K, respectively. However, the topographies obtained at the various temperatures, as shown in Figure 8, only slightly changed. The central heights of the residual ripple after unloading are 0.946, 1.026, 1.041, and 1.047 nm for 0, 200, 300, and 400 K, respectively.

**Figure 9:**
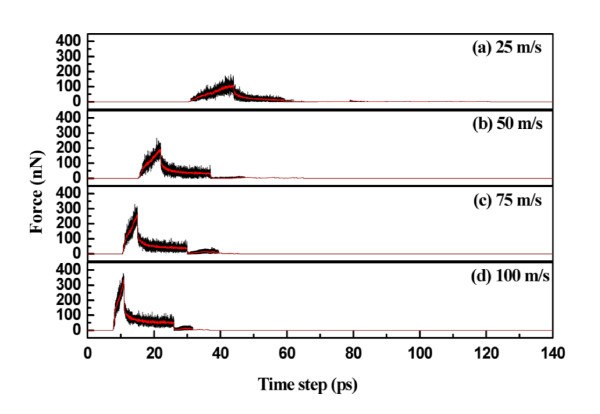
**Load versus time for various velocities**.

Indentation velocities of 25, 50, 75, and 100 m/s were tested by fixing the temperature at 300 K and the packing time at 15 ps. Figure 9 shows the load versus time curves for various indentation velocities. The load increases with increasing indentation velocity. This is due to the atoms having enough time to release and transfer internal residual stress at slower indentation velocities. The central heights of the residual ripple after unloading are 1.041, 0.907, 0.698, and 0.689 nm for indentation velocities of 25, 50, 75, and 100 m/s, respectively.

**Figure 10:**
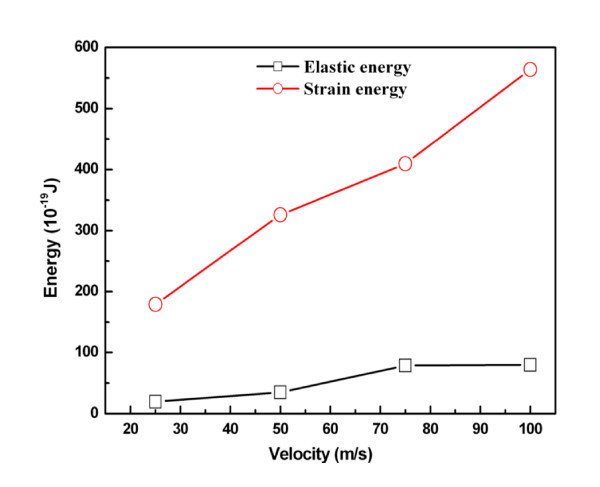
**Elastic energy and plastic energy versus velocity**.

**Figure 11:**
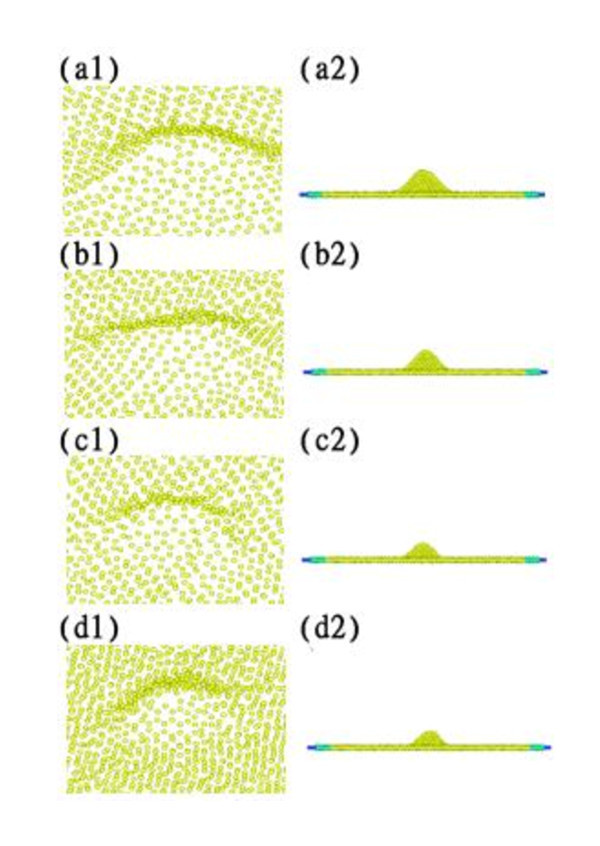
**Topographies for velocities**. Velocities of (a1, a2) 25 m/s, (b1, b2) 50 m/s, (c1, c2) 75 m/s, and (d1, d2) 100 m/s.

Figure 10 shows the elastic energy and plastic energy versus indentation velocity curves. Both energies increase with increasing velocity. Relaxation forces of 159.05, 222.93, 280.94, and 314.56 nN were obtained for indentation velocities of 25, 50, 75, and 100 m/s, respectively. Figure 11 shows the topographies obtained for various velocities. A slower indentation allows more atoms to adhere on the tip, and thus a larger area of the substrate is pull up during tip unloading, forming a higher peak.

## Conclusion

The indentation behavior of graphene was studied using molecular dynamics simulations. The following conclusions were obtained:

1. The affected area, load, elastic and plastic energies, and relaxation force increased with increasing indentation depth.

2. Nanoindentation-induced pile ups and corrugations of graphene were observed. Strong adhesion causes topological defects and vacancies.

3. The average contact stiffnesses of the graphene at temperatures of 0, 200, 300, and 400 K are 58.7, 58.1, 49.48, and 36.6 N/m, respectively.

4. At higher temperature, the kinetic energy among atoms increases, which weakens covalent bonds and thus decreases resistance to deformation. The load, elastic and plastic energies, and relaxation force decrease with increasing temperature.

5. With a fast indentation, the graphene has insufficient time to respond, which leads to a high load, elastic and plastic energies, and relaxation force.

## Competing interests

The authors declare that they have no competing interests.

## Authors' contributions

The work presented here was carried out in collaboration between all authors. THF, THW and JCY defined the research theme. THF and JCY designed methods and analyzed the data, interpreted the results and wrote the paper. YJH co-worked on associated data collection, their interpretation, and presentation. All authors have contributed to, seen and approved the final manuscript.
